# Selection and Characterization of Biofuel-Producing Environmental Bacteria Isolated from Vegetable Oil-Rich Wastes

**DOI:** 10.1371/journal.pone.0104063

**Published:** 2014-08-06

**Authors:** Almudena Escobar-Niño, Carlos Luna, Diego Luna, Ana T. Marcos, David Cánovas, Encarnación Mellado

**Affiliations:** 1 Department of Genetics, Faculty of Biology, University of Seville, Seville, Spain; 2 Department of Microbiology and Parasitology, Faculty of Pharmacy, University of Seville, Seville, Spain; 3 Department of Organic Chemistry, University of Córdoba, Córdoba, Spain; Universidade Nova de Lisboa, Portugal

## Abstract

Fossil fuels are consumed so rapidly that it is expected that the planet resources will be soon exhausted. Therefore, it is imperative to develop alternative and inexpensive new technologies to produce sustainable fuels, for example biodiesel. In addition to hydrolytic and esterification reactions, lipases are capable of performing transesterification reactions useful for the production of biodiesel. However selection of the lipases capable of performing transesterification reactions is not easy and consequently very few biodiesel producing lipases are currently available. In this work we first isolated 1,016 lipolytic microorganisms by a qualitative plate assay. In a second step, lipolytic bacteria were analyzed using a colorimetric assay to detect the transesterification activity. Thirty of the initial lipolytic strains were selected for further characterization. Phylogenetic analysis revealed that 23 of the bacterial isolates were Gram negative and 7 were Gram positive, belonging to different clades. Biofuel production was analyzed and quantified by gas chromatography and revealed that 5 of the isolates produced biofuel with yields higher than 80% at benchtop scale. Chemical and viscosity analysis of the produced biofuel revealed that it differed from biodiesel. This bacterial-derived biofuel does not require any further downstream processing and it can be used directly in engines. The freeze-dried bacterial culture supernatants could be used at least five times for biofuel production without diminishing their activity. Therefore, these 5 isolates represent excellent candidates for testing biofuel production at industrial scale.

## Introduction

With a growing world population, fossil fuels are currently consumed too rapidly. Thus, it is expected that we will deplete these non-renewable resources from the planet in a relatively short period of time. The increasing demand for fossil fuels has additional consequences, such as the concomitant increased prices of crude oil, the environmental concerns about the pollution due to crude oil derivatives and the global greenhouse effects, which altogether are triggering the exploration of novel alternative sources of fuels. Therefore, it is imperative to develop alternative, sustainable and inexpensive fuels. Biodiesel can be such a fuel with the appropriate technologies. Triglycerides (oils and fats) can not be directly used in the available diesel engines due to the high viscosity and the acidic composition of these lipids, the formation of free fatty acids resulting in gum formation by oxidation and polymerization, the carbon deposition and the thickening of the lubricant. Thus, the use of vegetable oils as alternative sources of fuels requires their processing to reach a viscosity and a volatility similar to crude oil derived fuels, a fact that will allow them to be directly used in the current configuration of diesel engines. These reasons are driving the development of new vegetable oil derivatives displaying properties similar to those of diesel fuels. Nowadays the most promising and accepted vegetable oil derivative is biodiesel [1], [2].

Biodiesel is a renewable source of energy considered to be carbon neutral because the carbon delivered during its combustion was fixed only a few years before from the atmosphere. In addition, its use in internal combustion engine does not produce sulfur oxide and minimizes three fold the formation of soot particulates in comparison with petrochemical diesel [3]. Biodiesel is a mixture of mono-alkyl esters that can be obtained from vegetable oils, animal fats, waste cooking oils, greases and algae. Three processing techniques are mainly used to catalyze the conversion of vegetable oils into ready-to-use biofuels: pyrolysis, microemulsification and transesterification. The most popular method is the transesterification (ethanolysis or methanolysis) of vegetable oils to produce biodiesel. The transesterification process can be performed using an alkali catalyst, an acid catalyst, a biocatalyst or heterogeneous catalysts, however only the alkaline process is currently carried out at industrial scale, because it is cost effective and highly efficient [4]. In the alkaline process sodium hydroxide (NaOH) or potassium hydroxide (KOH) is used as a catalyst along with methanol or ethanol. NaOH or KOH react with the alcohol to give an alcoxy group. Then the alcoxy moiety reacts with any triglyceride to form the corresponding methyl- or ethyl-esters and glycerol. After the synthesis, the production of biodiesel requires a series of downstream processes that include repeated washings to reach sufficient purity. These downstream operations are usually hampered with problems, such as the separation of catalyst and unreacted methanol from biodiesel, the risk of free fatty acid or water contamination and soap formation during the transesterification reaction [1], [2].

The production of biodiesel using a biocatalyst eliminates the disadvantages of the chemical alkaline process by obtaining a product of very high purity with less or no downstream operations. In particular, to avoid the need to separate glycerol from biodiesel, an alternative strategy based on incorporating some of the glycerol derivatives in the final product has been considered. In this way, a biofuel formed by a mix of glycerol derivatives and fatty acid methyl esters (FAME) or fatty acid ethyl esters (FAEE) not only prevents the need to separate the glycerol from the biodiesel, but also increases the yield of the process as the total number of carbons involved in the reaction is maintained in the final product [5], [6]. Some examples of methodologies considering this option are based on the transesterification reaction of triglycerides (TG) with dimethyl carbonate [7], methyl acetate [8] or ethyl acetate [9] resulting in a mixture of three molecules of FAME or FAEE and one of glycerol carbonate or glycerol triacetate. Another example is Ecodiesel-100, a patented biofuel obtained using 1,3-selective partial ethanolysis of triglycerides with a porcine pancreatic lipase. Ecodiesel-100 is a mixture of FAEE and monoglycerides (MG) [5], [6]. Despite of the advantages of Ecodiesel-100, the main drawback is the high cost of the purified enzyme. Therefore, there is a need to solve this issue before the process can be implemented at the industrial scale. The enzymatic production of biofuel is possible using extracellular or intracellular lipases.

Lipases (triacylglycerol acylhydrolases EC 3.1.1.3) are very versatile biocatalysts: they are stable in organic solvents; they do not require any cofactor; they have broad substrate specificity; and they show high enantioselectivity. The number of cloned lipases has increased since the 1980s, in part because of this versatility, but also because of the increased number of characterized lipolytic microorganisms. Several enzymatic activities have been described for lipases, such as hydrolysis, transesterification and esterification. However, the hydrolytic activity of lipases has been studied in detail in contrast to the transesterification activity. It is hypothesized that the transesterification activity of lipases is similar to the serine-protease catalytic activity, because both enzymes share the same catalytic triad Ser–His–Asp/Glu [10].

Extensive and persistent screening for new microorganisms and their lipolytic enzymes can open novel alternatives for synthetic processes and consequently, novel possibilities to contribute to solve environmental problems. The aim of this study is to find and select lipolytic microorganisms showing transesterification activity, which can be used to produce a cheap enzymatic extract, avoiding laborious downstream processing. Employing this enzymatic extract able to produce biofuel could reduce the cost of enzyme production. Therefore the selected microorganisms have the potential to be used in white biotechnology for the industrial production of a yet-to-be commercialized biofuel.

## Materials and Methods

### Site description and sample collection

Four samples were collected from different locations in an oil mill in Ecija (Sevilla, Spain) at the end of the harvesting season (January 2011). Pool 1 (AE1B) and pool 2 (AE2B) were sampled from ponds containing liquid wastes from the oil mill. Alpechín (AEA) was obtained from the solid wastes of the oil mill and the last sample (AEDH) was obtained from the wastes of the olive harvests containing mainly olive tree leftovers (i.e. dehydrated leaves and branches).

Samples were taken in sterile plastic 50 ml tubes and stored in the dark at 4°C until they were processed. The pH, electric conductivity (C.E.) and humidity of soil were measured as previously reported [11]. The extraction of bioavailable Ca, Mg, Na, K, Fe, Cu, Mn and Zn was performed as previously reported [12], [13]. Determination of metals was performed using atomic absorption spectrometry in a spectrophotometer UNICAM (Thermo). Determination of total C, N and S was performed in a LECO's CNS-2000 elemental autoanalyzer. Extraction and determination of bioavailable P was performed as previously reported [14], [15]. Determination of organic matter was done by calcination (UNE-EN 13039 standard) and the determination of total carbonates following the calcimetry protocol of Bernard [11].

### Isolation of microorganisms showing lipolytic activity (hydrolysis)

One gr of soil sample was resuspended in 3 ml of sterile saline solution (ClNa 0.85% w/v). This suspension was diluted with saline solution (ClNa 0.85% w/v) to obtain single colonies and plated on a battery of solid media containing 0.5% tributyrine. The media used were LB (Luria Bertani medium), Potato Dextrose Broth (PDB) (Difco), 2% Agar (w/v) in distilled water, 9K A (1 L 9K solution, 1% (w/v) glucose and 1% (w/v) yeast extract), 9K G (1 L 9K solution, 0.5% (w/v) glucose, 0.5% (w/v) yeast extract, and 1% (w/v) malt extract) or 9K Gamp (1 L 9K solution, 1% (w/v) glucose, 0.5% (w/v) yeast extract, 1% (w/v) malt extract and 20 µg/ml ampicillin). The 9K solution was prepared as described in Silverman [16] without FeSO_4_ 7H_2_0. To obtain solid media 2% agar was added to the media. The plates were incubated at 30°C for 4–7 days. Those microorganisms displaying a clearing zone around the colony were grown in plates containing non-selecting LB (bacteria), PDB (fungi) or YPD (yeast) media, and then the lipolytic activity was confirmed by growing the isolates again on the corresponding media containing 0.5% tributyrine.

### Screening for lipolytic microorganisms showing transesterification activity

For screening microorganisms displaying transesterification activity we developed a simple colorimetric method based on a previously reported one [17] with some modifications. The method consists on the transesterification of *para*-nitrophenyl palmitate (*p*-NPP) with ethanol in the absence of water to release the yellow colored compound *para*-nitrophenol (*p*-NP), which can be subsequently detected by using a spectrophotometer. A water-free environment promotes the transesterification reaction over the water-dependent hydrolisis reaction of *p*-NPP. A 5 ml overnight culture of each positive lipolytic bacterial isolate was employed to inoculate flasks containing 25 ml of LB media plus 2% tributyrine at a final OD_600_ of 0.1. These cultures were grown for 3 days at 30°C and 200 rpm. After this time cells were pelleted by centrifugation and 1.8 ml of the supernatants were freeze-dried for 24 hours. The freeze-dried supernatant was mixed with 1 ml of 10 mM *p*-NPP (in n-hexane) and 60 µl of absolute ethanol. The mixture was made in 2 ml safe-lock eppendorf tubes and incubated with shaking at 200 rpm in a rotator shaker at 37°C for 16 hours. A negative control was prepared by using a mixture of absolute ethanol and *p*-NPP. As a control of the hydrolytic activity of lipases we used a mixture of *p*-NPP and freeze-dried supernatant (without ethanol). After 16 hours of reaction the lipase was allowed to decant at the bottom of the tube for 10 minutes, then 25 µl of supernatant was mixed with 1 ml of 0.05 M NaOH in a 1.5 ml eppendorf tube. The *p*-NP produced during the transesterification reaction was extracted by the aqueous alkaline phase, transferred to a 1 ml cuvette. and the absorbance was quantified at 410 nm using a Beckman DU 640 spectrophotometer.

### Production of biodiesel at benchtop scale

The production of biofuel was analyzed by gas chromatography as previously reported [18]. A 5 ml overnight culture of each positive lipolytic bacterial isolate was employed to inoculate a flask containing 25 ml of LB media plus 2% tributyrine at a final OD_600_ of 0.1. Cultures were grown for 3 days at 30°C and 200 rpm. The whole volume of the supernatants was freeze-dried and all the extract was used directly in a transesterification reaction (non-processed supernatant). To eliminate salts and other components of the media and tributyrine from the supernatants they were concentrated in dialysis bags (12 KDa, Sigma) using polyethylene glycol (average Mw 8,000, Sigma) at 4°C overnight, then dialyzed in 0.05 M potassium phosphate buffer (pH 7.6) four times. The resulting concentrated and dialyzed supernatant was finally freeze-dried and used in a transesterification reaction (processed supernatant).

The transesterification reaction was performed with continous shaking at 37°C for 24 hours and contained 6 ml sunflower oil, 1.75 ml absolute ethanol, 0.05 ml NaOH 10N and the entire processed or non-processed supernatant coming from 25 ml of culture. A negative control was prepared by using a mixture of 6 ml sunflower oil, 1.75 ml absolute ethanol and 0.05 ml NaOH 10N. The ethyl esters and glycerides produced in the transesterification reaction were analyzed using gas chromatography (GC) as described below.

To study the kinetics of the reaction, samples were taken at the indicated time points, and analyzed by GC. To quantify the biofuel production after the re-utilization of the bacterial extracts, the reaction mix was centrifuged, the supernatant discarded and the pellet was employed to repeat the transesterification reaction and the GC analysis. Both analyses were performed using only processed supernatant.

### Chemical analysis of the biofuel

The method used integrates two official methods for the detection of esters (UNE EN ISO 14103) and glycerides (UNE EN ISO 14105), using cetane (n-hexadecane) as an internal standard to quantify the contents of glycerol, ethyl esters and glycerides (mono-, di- and triglycerides) as previously described [5]. Briefly, a gas chromatograph Varian 430 GG fitted with a capillary column HT5, 0.1 µm (25 m×0.32 mm, SGE, Supelco) with a flame ionization detector (FID) and *splitless* injection was used. The transesterification reaction product (12.5 µl) was mixed with 4 ml of a 1∶1 (v/v) ethanol/dichloromethane mixture that contained the internal standard (cetane), and 0.5 µl of the prepared sample was employed for the analysis.

The results were expressed as relative quantities of the corresponding fatty acid ethyl esters (FAEE) and some monoglycerides (MG) of lower retention time (RT<25 minutes) and the sum of the quantities of the other MG (RT>25 minutes) and the diglycerides (DG). The yield refers to the relative amount (%) of FAEE + MG (with lower RT) produced. The conversion includes the total amount (%) of triglyceride transformed into FAEE, MG and DG. Blank reactions containing only the mixture of oil, ethanol and NaOH were performed. Conversion of the starting oil material was below 15% under these experimental conditions. Data shown are the average of at least two independent experiments.

### Determination of the viscosity

The viscosity was determined in a capillary viscometer Oswald Proton Cannon-Fenske Routine Viscometer 33200, size 2150. The method used was based on determining the time needed for a given volume of fluid to pass between two points marked in the instrument. It correlates with the speed reduction suffered by the liquid flow as a result of internal friction of its molecules, depending on their viscosity. From the flow time, *t*, in seconds, the kinematic viscosity (*υ*, centistokes, cSt) can be obtained from the equation: *υ*×*t* = *C*, where *C* is the constant calibration of the measuring system in cSt·s, which is given by the manufacturer (0.10698 mm^2^ s^−^1, at 40°C) and t the flow time in seconds. The kinematic viscosity also represents the ratio between the dynamic viscosity and the density (*ρ*, *υ* = *η*/*ρ*).

### Analysis of hydrolytic activities (amylase, protease, DNAse, pullulanase and xylanase)

Amylase activity was screened on LB solid medium supplemented with 0.2% (w/v) soluble starch, 0.5% (w/v) peptone and 0.3% (w/v) meat extract. After 7 days of incubation at 30°C the plates were flooded with 0.3% (w/v) I_2_–0.6% (v/v) KI solution. Hydrolysis of starch results in a clearing zone around the colonies [19].

The presence of protease activity was determined in LB solid medium supplemented with 2% (w/v) skim milk. The appearance of zones of precipitation of paracasein around the colonies after 3 days of incubation at 30°C indicated the presence of proteolytic activity [19].

DNAse activity was analyzed by growing bacteria on DNAse test agar plates containing 4.2% (w/v) Agar DNA. The plates were incubated for 7 days at 30°C and then flooded with 1N HCl solution. A clearing zone around the colonies indicated DNase activity [20].

The pullulanase and xylanase activities were detected by screening for zones of blue halos produced due to the hydrolysis and solubilization of the AZCL-pullulano and AZCL-xylano, respectively [21].

### Isolation of DNA and 16S rRNA gene sequence analysis

The bacterial DNAs were isolated following standard protocols [22]. The total DNA isolated was used as the template for the amplification of the 16S rRNA by PCR using the universal primers designed for Bacteria 16F27 (5′-AGAGTTTGATCMTGGCTCAG-3′) and 16R1488 (5′-CGGTTACCTTGTTAGGACTTCACC-3′) [23]. The program used for the amplification was: one cycle of 95°C for 5 minutes; 25 cycles of 94°C for 1 minute, 50°C for 1 minute and 72°C for 2 minutes; and a final extension cycle for 10 minutes at 72°C. Partial 16S rRNA gene sequences (c. 650 bp corresponding to positions 39 to 689 of the 16S rRNA gene from *Escherichia coli*) were obtained and aligned to the most similar 16S rRNA gene sequence in the GenBank database using the BLASTn algorithm. Using the most similar sequences found in the GenBank database a multiple sequence alignment of the DNA sequences was constructed using the ClustalW software [24]. The phylogenetic trees were obtained using the MEGA4 software [25] with the neighbor-joining method [26]. The data set was bootstrapped 500 times to ensure reliability of each branch. The evolutionary distances were computed using the maximum composite likelihood method [27] and are shown in number of base substitutions per site.

### Nucleotide sequence accession numbers

The nucleotide sequences reported in this work have been deposited in the GenBank database under accession numbers KC880159 to KC880188.

## Results and Discussion

### Chemical composition of the collected samples

One of the aims of this work was to find microorganisms capable of producing biofuel from vegetable oil. Therefore, locations rich in vegetable oil were selected to screen for microorganisms harboring lipase activity against vegetable oil. In particular, these samples were collected from an olive oil mill located in Ecija, Southern Spain.

The chemical and physico-chemical characteristics of the collected samples are shown in Table 1. The analysis of the samples revealed that the most abundant metal element in all samples was Fe, although in the sample AEDH there were only minor differences between the Fe, Mn, Cu and Zn content. The rest of elements analyzed were C>Ca>N>K>S>Mg>Na>P (from most to less abundant). The exceptions were sample AE2B where S was no detected, sample AEA where P was more abundant than Na, and AEDH where Mg was more abundant than S. The pH of all samples was around 6.5, except in AEA (*alpechín*), which showed pH 4.7. In most of the samples the organic material accounted for 30–40% except for the AEA, which was the richest one with a 95.8%.

**Table 1:** Chemical and physico-chemical analysis of the oil mill samples.

Parameter			Samples^a^	
	AE1B	AE2B	AEA	AEDH
**C total (%)**	16.24	22.490	66.410	22.970
**N total (%)**	0.894	0.616	1.169	0.819
**S Total (%)**	0.05594	N.D	0.100	0.052
**K (%)**	0.144	0.110	0.456	0.269
**Na (mg/Kg)**	61.234	130.597	168.019	235.668
**Mg (%)**	0.018	0.030	0.022	0.059
**Ca (%)**	1.602	1.500	0.074	1.861
**P (mg/Kg)**	36.755	43.541	280.390	168.848
**Mn (mg/Kg)**	63.528	95.033	12.015	125.528
**Fe (mg/Kg)**	599.663	651.608	139.812	151.260
**Cu (mg/Kg)**	73.277	49.632	27.195	140.325
**Zn (mg/Kg)**	19.163	14.253	29.750	11.955
**pH (1/5)**	6.837	6.55	4.68	6.27
**E.C. 1/5 (mS/cm)** b	1.68	2.62	3.89	2.14
**Humidity (%)**	5.460	4.690	5.819	5.179
**O.M. (%)** c	30.709	40.186	95.801	40.782
**CaCO_3_ total (%)**	9.499	8.808	1.262	6.314

a All the results are referred to the dried mass of the samples.

b E.C.: Electric Conductivity.

c O.M.: Organic Matter.

### First step of the screening: selection of microorganisms showing lipolytic activity (hydrolysis)

As a first step of the screening process, we assayed for the hydrolytic activity of the microorganisms. This is an easy, quick and cheap test to perform on solid media based on the visual inspection of plates containing the lipid substrate tributyrine for microorganisms showing a clearing zone around the colony edges. To obtain single colonies and select lipolytic microorganisms, samples were diluted in sterile saline solution and plated on LB, PDB, water-agar, K9 A, K9 G or K9 Gamp solid media supplemented with 0.5% tributyrine. During the first round of selection, 1,016 colonies (fungi, yeasts and bacteria) were identified to produce a clearing zone of hydrolysis (Table 2). Most microorganisms were obtained from media K9 A, K9 G and K9 Gamp (68% fungi, 65% bacteria, 65% yeasts). We decided to study only the lipolytic bacteria and thus, we selected 291 bacteria for further studies. These strains were first inoculated on non-selective media and re-checked for hydrolytic activity on LB supplemented with 0.5% tributyrine. Sample AEA yielded the poorest number of lipolytic bacteria. The AEA sample was obtained from *alpechín*, which is the toxic wastewater resulting after pressing the olives during the production of oil. The analysis of this sample showed that it was acid and rich in organic matter. This result is in agreement with previous reports describing that *alpechín* is slightly acid and rich in soluble organic compounds [28], [29]. Although AEA was the sample containing the highest organic material content, usually lignin accounts for ca. 50% of the total organic material in this type of samples. Lignin is a recalcitrant polymer that may also affect microbial activity and survival negatively. In fact, the phytotoxic and antimicrobial effects of phenols, organic acids and fatty acids that are usually present in this wastewater *alpechín* have been previously reported [30]. This may explain the low number of lipolytic microorganisms found in this sample.

**Table 2:** Distribution of types of lipolytic microorganisms isolated from the oil mill samples.

Sample	Bacteria	Yeast	Fungi	Total
**AE1B**	61	21	174	256
**AE2B**	166	208	10	392
**AEDH**	57	190	1	248
**AEA**	7	70	51	128
**Total**	291	489	236	**1016**

### Second step of the screening: Analysis of the transesterification activity of lipolytic microorganisms

We have employed a method based on the transesterification of *p*-nitrophenolpalmitate (*p*-NPP) with ethanol (or other alcohol) to release the yellow-colored *p*-nitrophenol (*p*-NP) as described in material and methods. In order to select only extracellular enzymes, this second step of the screening was performed using freeze-dried supernatant of the bacteria selected in the first step of the screening.

During the screening procedure, a diverse set of control assays was carried out in the absence of ethanol or lipolytic bacteria. The *p*-NP released under these control conditions was considered to result from the hydrolysis or a non-enzymatic reaction of *p*-NPP and it was employed as the threshold to determine the existence of transesterification activity. The maximum absorbance value obtained in the control reactions was 0.8. The transesterification reaction with all the strains selected during the first step of the screening was performed in parallel. All strains showing absorbance values over this threshold of 0.8 were considered positive and values below the threshold were considered background levels (Table 3). According to the above criterion, 58 strains were considered positive. Out of the 58 positive strains, the top 30 bacteria, i.e. those showing absorbance values over 1.0, were selected for further analysis and quantification of biofuel production.

**Table 3:** Classification and selection of bacteria showing transesterification activity according to the *para*-nitrophenylpalmitate test.

Absorbance 410 nm	Number of strains	Positive/Negative
>1	30	Positive
1–0.8	28	Positive
<0.8	233	Negative

During this step we selected only for bacteria showing extracellular transesterification activity because it is described that at industrial level the highest yields of biodiesel production are obtained with extracellular lipases rather than with whole cells [1], [3]. In fact, whole cell transesterification procedures produce low substrate conversion yields due to the toxicity of the solvent to the host cells, and also due to the low mass transfer rate of high molecular weight substrates (oil) from the solvent phase to the whole cell biocatalyst [31]. Another noticeable difference when the transesterification reaction is performed with whole cells is the reaction time. Novozym 435 gave yields of 87% after 3.5 h of reaction in a fed-batch operation. However, to obtain yields of 80–90% of biodiesel using whole cells, it was required to perform the reaction process for 70 h [32].

### Third step of the screening: Selection of the bacterial supernatants producing biofuel at benchtop scale

**Figure 1:**
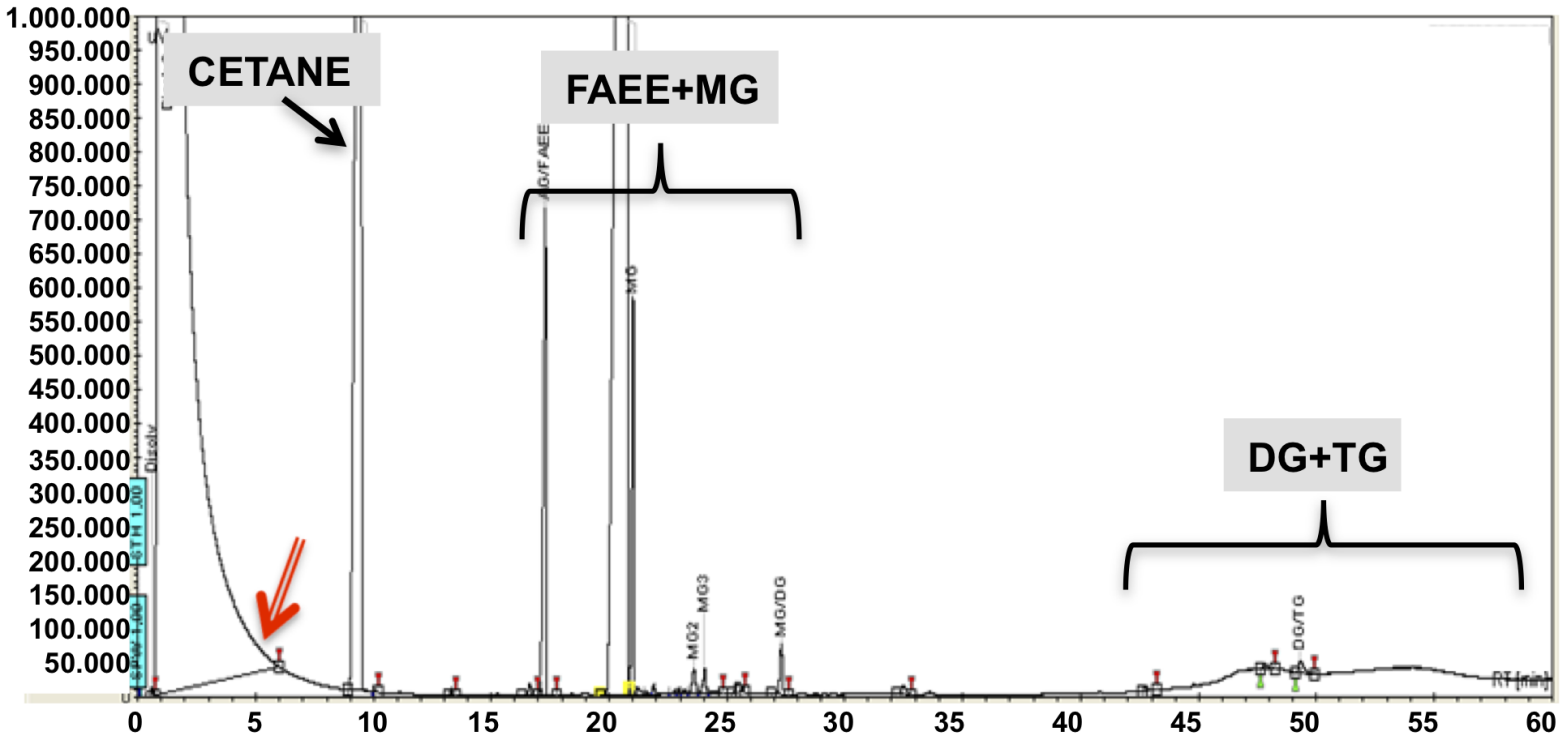
Chromatogram obtained by GC of the reaction mix after the transesterification of sunflower oil and ethanol performed by the processed supernatant of strain AE2B 122. Cetane was used as an internal standard. FAEE: Fatty Acid Ethyl Ester; MG: MonoGlyceride; DG: DiGlyceride; TG: TriGlyceride. The double-lined arrow indicates the retention time expected for glycerol as previously reported [5], [6].

**Figure 2:**
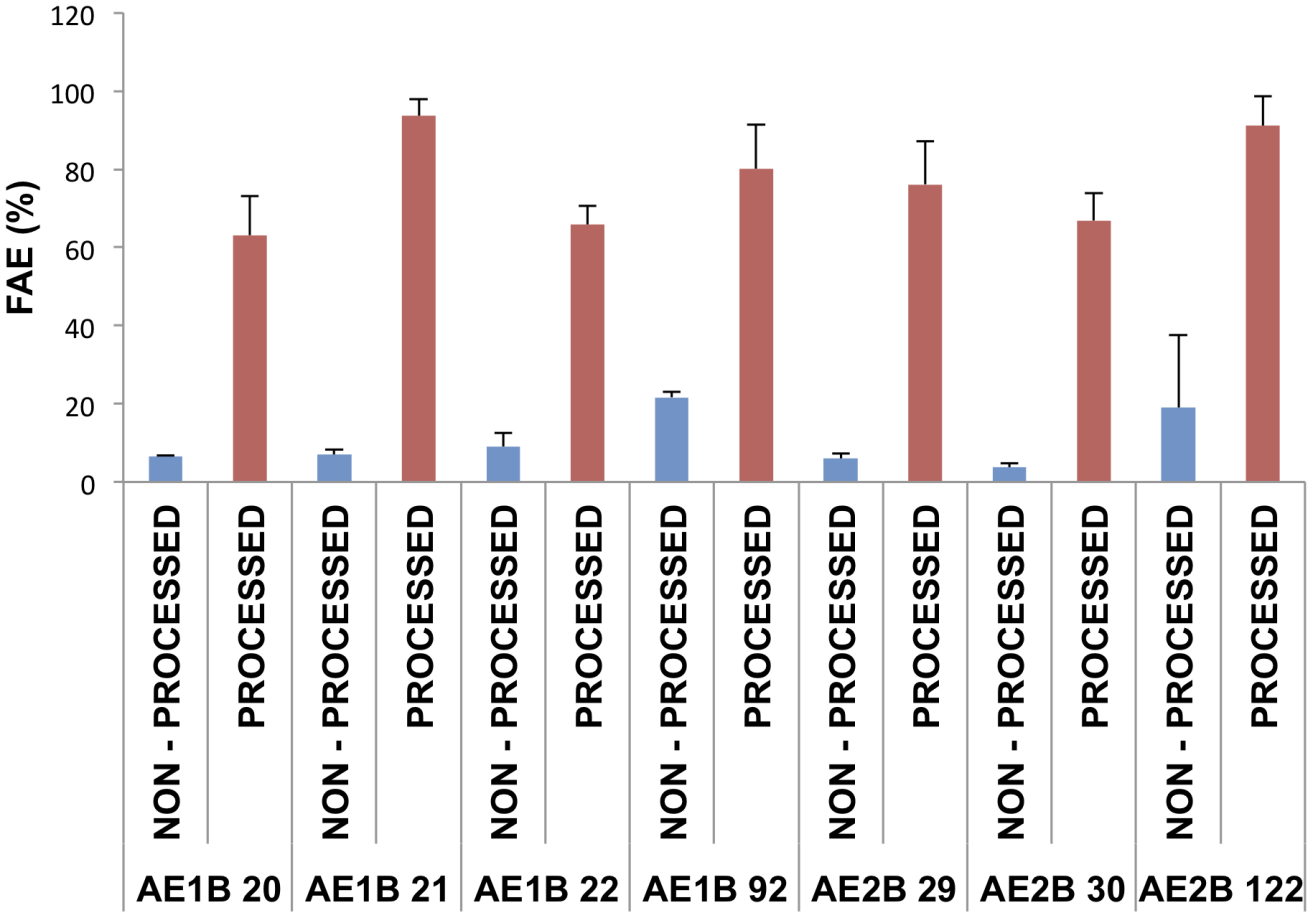
Comparison of biofuel production using processed or non-processed supernatants. The bacterial supernatants were processed as described in Materials and Methods or not processed. Both processed and non-processed supernatants were freeze-dried and employed for transesterification reaction with sunflower oil and ethanol. The data show the percentage of FAE (FAEE+MG) produced after 24 hours of reaction using 7 randomly selected bacterial isolates. Data are the average of at least 2 independent experiments.

The aim of the third step of the screening was to identify the best performing bacteria in biofuel production at benchtop scale. To optimize the screening protocol, we first grew the 30 selected bacterial strains as described in Materials and Methods. In a first attempt the supernatants (non-processed) were freeze-dried to obtain a fine dried powder that can be used directly for the production of biofuel. The transesterification reaction to produce biofuel was performed at basic pH with sunflower oil, ethanol and the powdered supernatant. After completion of the reaction, the biofuel/lipid/fatty acid mixture was analyzed by GC. The chromatograms show clear different retention times for the final (FAEE + MG), and the initial (TG) and intermediate (DG) products (Fig. 1). The yield obtained with the bacteria initially tested was lower than 22% in all cases (Fig. 2). In order to try to increase the yield, the supernatants of the cultures were processed as described in materials and methods, and the samples were then freeze-dried and assayed for biofuel production. We observed that processing of the bacterial supernatants drastically increased the performance up to 17.8 fold in comparison to the non-processed samples (Fig. 2). Therefore, we found out that an easy and cheap process of concentration and dialysis could increase the yield of biofuel production. Once the protocol was optimized, we proceeded to quantify biofuel production in the 30 selected bacterial strains. The complete results of the reactions using processed supernatants are shown in Table 4. Since the composition of biodiesel is a mix of FAEEs and the composition of biofuel is a mix of FAEs, the most important value to be considered is the percentage of FAE, which are a mix of two moles of FAEE and one mol of MG. The analysis of the data revealed that the supernatants of 5 of the bacterial strains were capable of producing over 80% FAE, 9 bacterial supernatants produced between 60–80% FAE, and the production of the other 16 isolates was below 60% FAE. Eight bacterial strains were capable of processing over 80% of the TG in the reaction mixture (conversion of TG into DG plus FAE).

**Table 4:** Transesterification reaction of sunflower oil by the bacterial extracts determined by GC analysis.

Sample	*FAE %* a	*DG %*	*TG %*	*Conversion %*
**Sunflower oil**	3.4	29.9	66.7	33.3
**Negative Control**	13.8	41.9	44.3	55.7
**AE1B 20**	63.1	6.7	30.2	69.8
**AE1B 21**	93.7	6.3	0.0	100
**AE1B 22**	65.9	2.5	31.7	68.4
**AE1B 26**	80.1	12.9	7.1	92.9
**AE1B 27**	57.9	4.3	37.7	62.3
**AE1B 28**	58.7	3.1	38.2	61.8
**AE1B 35**	67.4	2.5	30.2	69.8
**AE1B 89**	72.2	11.6	16.2	83.8
**AE1B 90**	58.6	2.7	38.7	61.3
**AE1B 92**	80.1	7.5	12.4	87.6
**AE2B 29**	76.0	7.8	16.2	83.8
**AE2B 30**	66.8	5.1	28.1	71.9
**AE2B 85**	41.2	13.8	45.1	54.9
**AE2B 120**	35.1	15.1	50.1	50.2
**AE2B 122**	91.16	1.75	6.8	93.2
**AE2B 130**	71.8	8.1	20.1	79.9
**AE2B 131**	61,27	9,55	14,59	85,41
**AE2B 133**	34.8	19.5	45.7	54.3
**AE2B 134**	31.7	2.1	66.3	33.7
**AE2B 199**	25.0	3.3	71.7	28.3
**AE2B 222**	77.5	3.2	12.9	87.1
**AE2B 232**	48.1	0.7	51.2	48.8
**AE2B 250**	46.4	10.7	42.9	57.1
**AE2B 259**	30.7	8.3	61.0	39.0
**AE2B 261**	82.6	7.8	9.6	90.4
**AE2B 263**	49.3	10.5	40.2	59.8
**AE2B 264**	24.1	2.6	73.3	26.7
**AE2B 332**	54.0	0.7	45.4	54.7
**AE2B 340**	51.4	1.0	47.6	52.4
**AEDH 145**	61.0	4.0	35.0	65.0

The percentage of FAE shows the yield of biofuel production in the reaction mix. Conversion shows the percentage of TG metabolized during the reaction.

a FAE, Fatty Acid Esters (fatty acid ethyl esters + monoglycerides) (Biofuel). DG, Diglycerides. TG, Triglycerides. Conversion, percentage of TG converted into FAE+DG.

During the screening for the best biofuel-producing strains, we noticed that glycerol did not appear at the expected retention time (5 min). This suggests that TGs were converted into ethyl esters (FAEEs) and MG, which co-elute under our experimental conditions (Fig. 1). This was previously observed in transesterification reactions performed using porcine pancreatic lipase [6]. From an industrial perspective, the biofuel produced by these bacteria would not require downstream processing to separate the glycerol contaminant because glycerol was integrated in the final mix as MG. This could be due to a 1,3 selective ethanolysis of the sunflower oil by the enzyme, that means a partial ethanolysis of the oil that produce 1 mol of MG for each 2 moles of FAEE [6]. The chemical analysis also suggests that this methodology is not suitable to produce conventional biodiesel according to EN 14214, but rather the new type of biofuel, which incorporates glycerol as previously described [5], [6].

### Phylogenetic analyses of the selected biofuel producing bacteria

**Figure 3:**
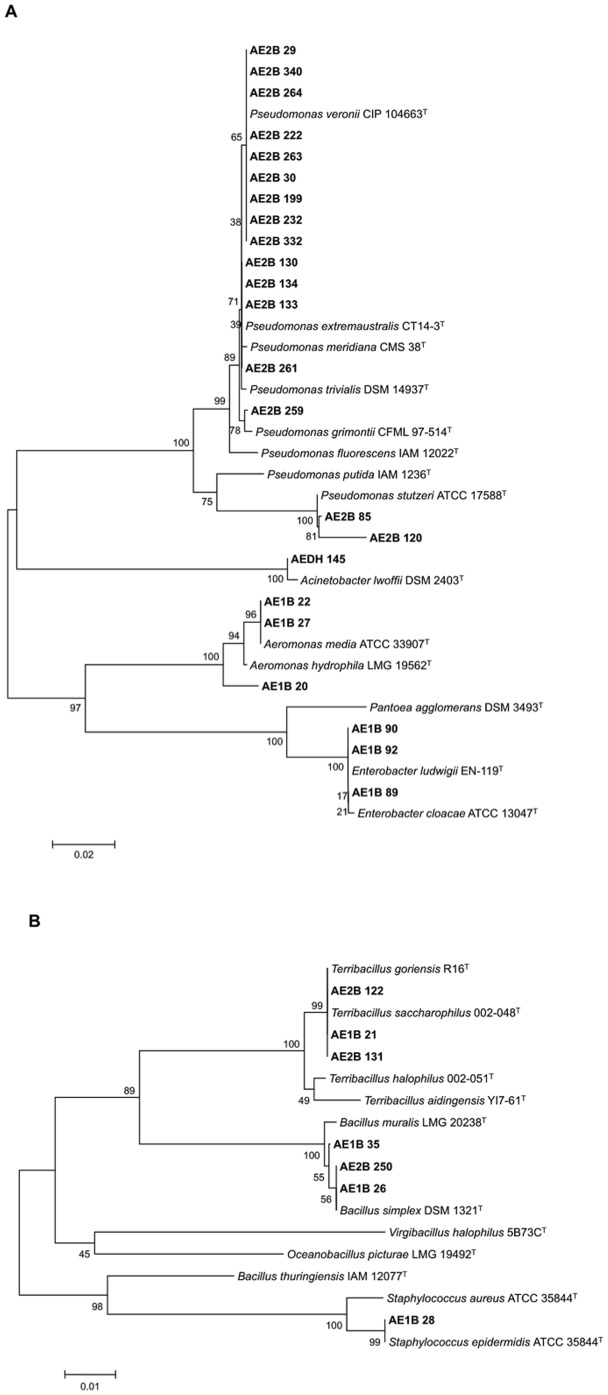
Evolutionary relationships of the selected strains. Phylogenetic trees were inferred from the 16S rRNA sequences of the 23 Gram negative (A) and the 7 Gram positive (B) bacteria with Neighbor-Joining clustering. The distances were calculated using Maximum Composite Likelihood. The bacterial strains isolated in this work are indicated in bold. 16S rRNA gene sequences from the isolates correspond to 650 bp. Bar represents a 2% (A) or 1% (B) of sequence difference.

In order to assign the bacterial biofuel producers to specific phylogenetic groups, the sequence of the 16S rRNA gene was determined. Phylogenetic reconstruction performed with different methods was consistent, and consequently only the tree obtained with Neighbor-Joining for the evolutionary history and Maximum Composite Likelihood for evolutionary distances is shown (Fig. 3). The strains isolated were very diverse with representatives of both Gram positive and Gram negative bacteria. This analysis together with the high number of eukaryotic microorganisms (yeasts and fungi) also isolated during this work suggests that there is a high diversity of living organisms in the samples of the olive-mill wastes. Table 5 shows the closest relative to each of the isolates according to the results obtained after the phylogenetic analysis.

**Table 5:** The closest relative of each isolate is indicated based on the phylogenetic reconstruction shown in Figure 3.

Sample	Closest relative	Accession number	% Similarity
**AE2B 29 AE2B 30 AE2B 199 AE2B 222 AE2B 232 AE2B 263 AE2B 264 AE2B 332 AE2B 340**	*Pseudomonas veronii*	AF064460	99–100
**AE2B 130 AE2B 133 AE2B 134 AE2B 261**	*Pseudomonas extremaustralis*	AJ583501	99–100
**AE2B 259**	*Pseudomonas grimontii* CFML 97-514T; AF268029	AF268029	99
**AE2B 85 AE2B 120**	*Pseudomonas stutzeri*	AF094748	98–100
**AEDH 145**	*Acinetobacter lwoffii*	X81665	99
**AE1B 89 AE1B 90 AE1B 92**	*Enterobacter ludwigii*	AJ853891	99–100
**AE1B 20**	*Aeromonas hydrophila* strain: LMG 19562; AJ508765	AJ508765	99
**AE1B 22 AE1B 27**	*Aeromonas media*	X60410	99–100
**AE1B 26 AE1B 250**	*Bacillus simplex*	AJ439078	99–100
**AE1B 35**	*Bacillus muralis*	AJ316309	99
**AE1B 28**	*Staphylococcus epidermidis* ATCC 14990; D83363	D83363	99
**AE1B 21 AE2B 122 AE2B 131**	*Terribacillus goriensis* a *T. saccharophilus* a	DQ519571 AB243845	99–100

Samples AE1B and AE2B were obatined from ponds containing liquid wastes from the oil mill. Sample AEDH was obtained from the wastes of the olive harvests containing mainly olive tree leftovers (leaves and branches).

a Strains AE1B21, AE2B122 and AE2B131 showed the same % of similarity to both *T. goriensis* and *T. saccharophilus*.

Most of the lipolytic isolates were obtained from the sample AE2B (19 isolates): 16 of them were related to the genus *Pseudomonas*, 2 of them related to the genus *Terribacillus*, and one isolate related to the genus *Bacillus*. Eleven lipolytic isolates were obtained from the sample AE1B, which were related to the genera *Enterobacter* (3 isolates), *Aeromonas* (3 isolates), *Bacillus* (2 isolates) and *Terribacillus* (one isolate). Only one isolate was obtained from the sample AEDH. It was related to the genus *Acinetobacter*. Interestingly, no lipolytic isolates were selected from the sample AEA (Table 5).

The isolates that showed the highest yield in the production of biofuel (selectivity over 60%) were 14 strains belonging to the genera *Pseudomonas*, *Acinetobacter*, *Enterobacter*, *Bacillus*, and *Terribacillus* (Table 4 and 5). Their closest relatives were *Pseudomonas veronii* [33] (AE2B 30, AE2B 29, AE2B 222), *Pseudomonas extremaustralis* [34] (AE2B 130, AE2B 261), *Acinetobacter lwoffii* [35] (AEDH 145), *Enterobacter ludwigii* [36] (AE1B 89 and AE1B 92), *Bacillus simplex* [37] and *B. muralis* [38] (AE1B 26 and AE1B 35), *Aeromonas hydrophila* and *A. media* (AE1B 22 and AE1B 20), and *Terribacillus goriensis* [39] and *Terribacillus saccharophilus* [40] (AE1B 21 and AE2B 122). Several of the selected isolates belong to genera that have been already reported to express transesterification activity, e.g. *Pseudomonas, Enterobacter* and *Bacillus*. Other microorganisms that have been reported to have enzymes with transesterification activity are *P. fluorescens, P. cepacia, Rhizomucor miehei, Rhizopus oryzae, Candida rugosa, Thermomyces lanuginosus, Candida antarctica, Chromobacterium viscosum, Burkholderia cepacia, E. aerogenes, Mucor miehei, Penicillium expansum* and *B. subtilis* [31]. It is interesting to note that the strains AE2B 122 and AE1B 21 showing the best performance in the production of biofuel under our experimental conditions belong to the genus *Terribacillus*. To the best of our knowledge there are no reports on the employment of members of the genus *Terribacillus* for transesterification reactions, and therefore, they constitute novel producers of biofuel synthesizing enzymes.

### Viscosity measurements

**Figure 4:**
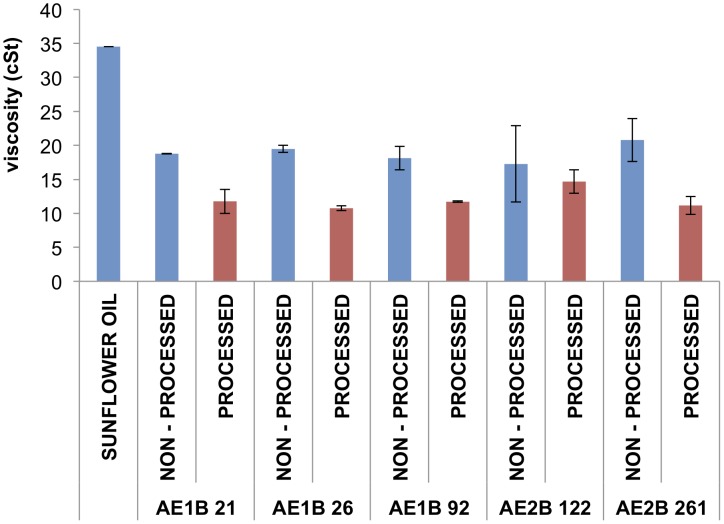
Viscosity of the sunflower oil, and the transesterification product using processed and non-processed supernatants of selected bacteria. Biofuel (FAEE+MG) was obtained by transesterification reaction of sunflower oil and ethanol with processed or non-processed supernatants of the best 5 biofuel-producing bacterial strains. Data are the average of at least two independent experiments.

The high similarity of the chromatography retention values obtained between different MG and FAEE compounds suggests that the rheological properties of these compound mixes were similar. This is an important factor for future applications as biofuel in diesel engines. Therefore, we measured the viscosity of the bacteria-derived biofuel, as a critical parameter for employing the biofuel. Figure 4 shows the viscosity of the supernatants of the top 5 biofuel-producing bacteria. All bacterial supernatants selected for biofuel production reduced the viscosity of the reaction product at least three times compared to the viscosity of the sunflower oil. Processing the bacterial supernatants as described in materials and methods further reduced the viscosity of the reaction product (Fig. 4), providing another evidence of the advantage of employing processed supernatants over non-processed supernatants.

### Kinetics of biodiesel production and re-utilization of bacterial supernatants

**Figure 5:**
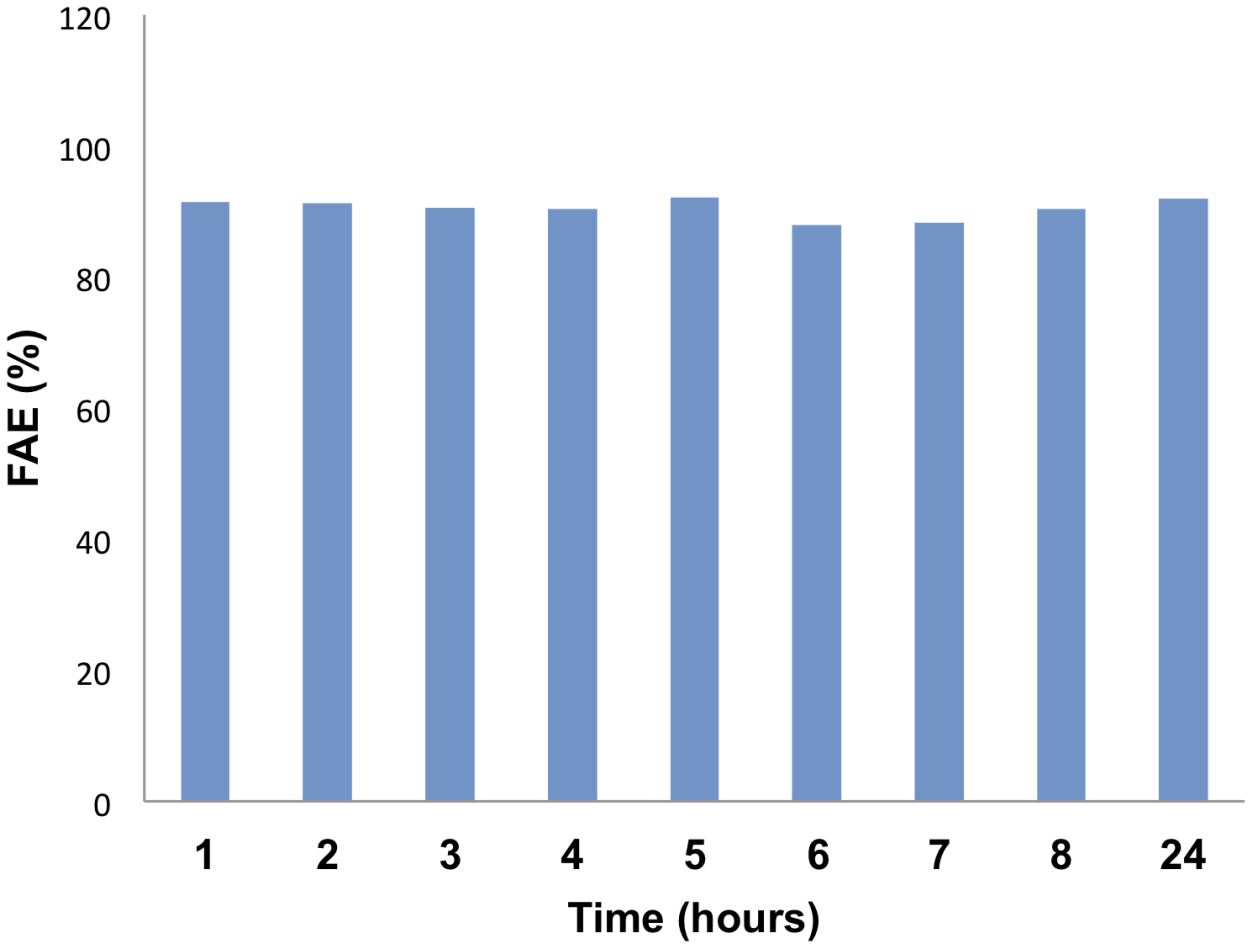
Kinetic analysis of the production of biofuel. The transesterification reaction of sunflower oil and ethanol by processed supernatants was set up, and samples were withdrawn and analyzed by GC at the indicated time points. The experiment was performed with 3 different bacterial strains. The data show one representative experiment of the kinetic analysis performed with *Enterobacter* sp. AE1B 92 supernatants.

All previous experiments of biofuel production were performed for 24 h. Three strains were randomly selected to test whether the reaction times could be shorten under our experimental conditions. The reaction was performed with processed supernatants as described above for 24 h and samples were withdrawn at the indicated time points to analyze the chemical composition of the reaction mixture. Figure 5 shows a representative example of a kinetic experiment performed with strain *Enterobacter* sp. AE1B 92. After one hour of reaction, the bacterial supernatant was capable of producing over 90% of FAE. There was slight variation in the reaction composition over the 24 h period of incubation. Similar results were found with two other bacterial strains (data not shown).

**Figure 6:**
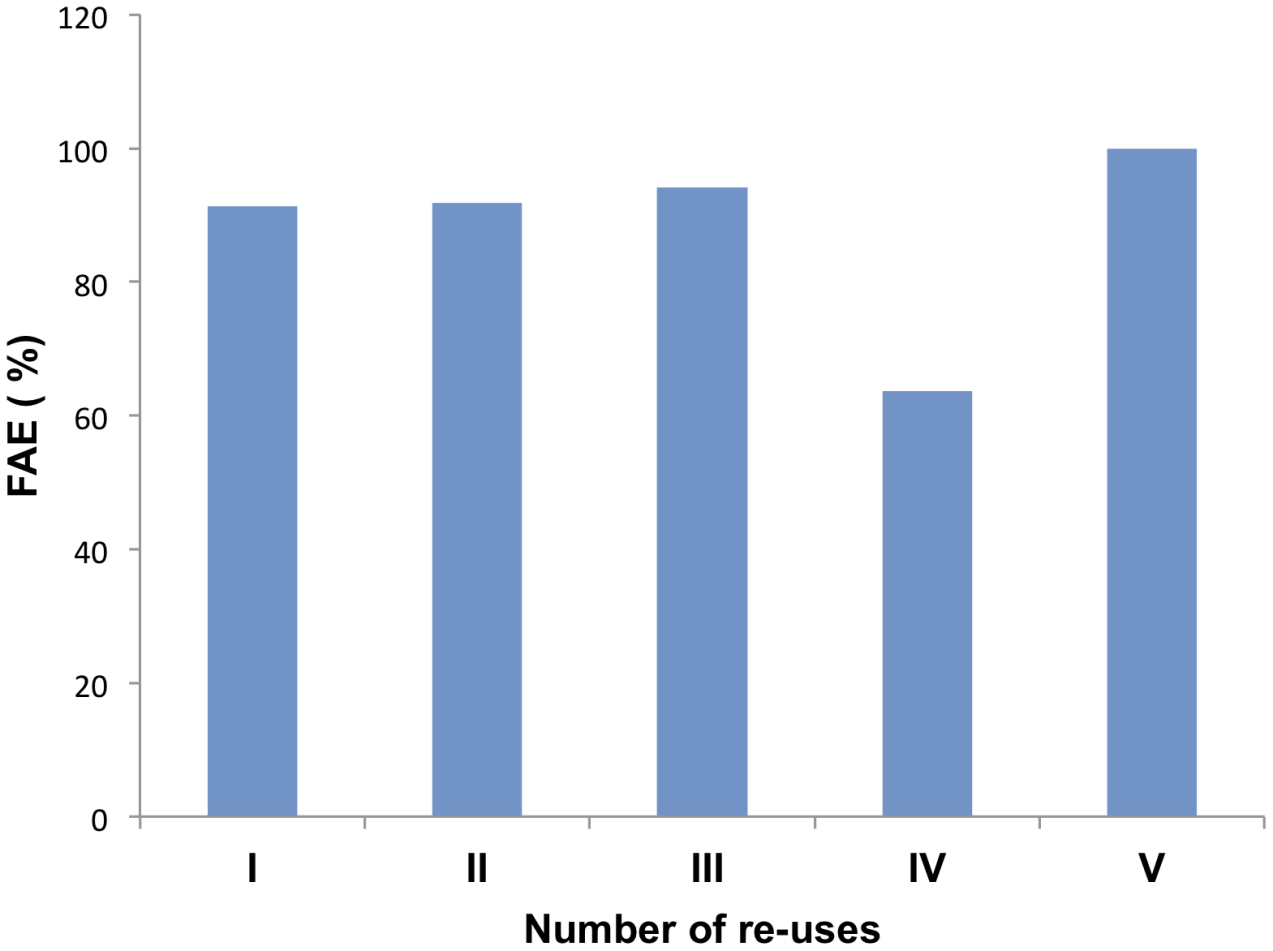
Re-utilization of bacterial supernatants for the production of biofuel. The transesterification reaction of sunflower oil and ethanol was performed with processed supernatants of the bacterial strains. After completion of the reaction, the mix was centrifuged, and the biofuel was analyzed by GC. The pellet was employed for another transesterificaction reaction. The reaction was repeated 5 times with the same extracts. The experiment was performed with 5 different bacterial strains. The data show one representative experiment performed with *Enterobacter* sp. AE1B 92 supernatant.

An economical factor to be considered during the industrial process affects the number of times that a supernatant can be employed during the biofuel production. Therefore, we randomly selected five bacterial strains to study the number of times that they can be employed. In all cases, the supernatants could be employed at least five times without losing their activity significantly (Fig. 6 shows a representative experiment of the 5 strains).

### Characterization of the hydrolytic activities of the selected biofuel-producing bacteria

To further characterize the biofuel-producing bacteria, we analyzed the hydrolytic activities of the 30 strains. All the bacterial strains were tested in LB plates containing the corresponding substrates. The amylase activity was the most abundant hydrolytic activity found in these isolates, as 11 isolates showed amylase activity. Ten bacteria showed DNAse activity, 9 showed protease activity and 5 showed pullulanase activity. However, none of them showed xylanase activity (Table S1 in File S1). Three of the isolates (*Aeromonas* sp. AE1B 27, *Aeromonas* sp. AE1B 22 and *Aeromonas* sp. AE1B 20) showed all the hydrolytic activities (except xylanase). There were 12 bacteria that did not show any hydrolytic activity under our assay conditions, which accounts for 40% of the isolates.

## Conclusion

In this work we describe the selection and isolation of microorganisms able to perform a transesterification reaction. We have employed a three-step screening to isolate lipolytic microorganisms able to produce a biofuel, consisting of a mix of FAEE and MG with lower viscosity than sunflower oil. Five of the bacterial strains isolated were capable of producing biofuel with yields higher than 80%. The best two performing strains belong to the genus *Terribacillus*, which has not been reported as biofuel producer so far. These two strains can be of great interest at industrial scale. In addition, here we report an easy and cheap method for processing the bacterial supernatant, which is capable of producing biofuel efficiently without further downstream processing or the need for expensive protein purification.

## Supporting Information

File S1 **Characterization of the hydrolytic activities of the selected biofuel-producing bacteria and Table S1.**
(PDF)
