# Supplementary material for: Selection and Characterization of Biofuel-Producing Environmental Bacteria Isolated from Vegetable Oil-Rich Wastes
Source: PLoS One. 2014 Aug 6;9(8):e104063. doi: 10.1371/journal.pone.0104063 (PMC4123985; doi:10.1371/journal.pone.0104063)
Supplement: File S1 — Characterization of the hydrolytic activities of the selected biofuel-producing bacteria and Table S1. (PDF) [file pone.0104063.s001.pdf]

SUPPLEMENTARY MATERIAL

Characterization of the hydrolytic activities of the selected biofuel-producing bacteria

After comparing the hydrolytic data with the phylogenetic reconstruction (Table 5 and Table S1), it became evident that most of the amylase producers belong to the genus *Pseudomonas* (7 out of 11 strains). 5 out of the 10 DNAse producers also belong to the genus *Pseudomonas,* and 3 strains to the genus *Aeromonas*. The group of protease producers comprised 9 strains and it was more diverse than the group of amylase or DNAse producers: 4 of them belong to the genus *Pseudomonas*, 3 to the genus *Aeromonas* and 2 to the genus *Bacillus*. The group of pullulanase producers included 2 strains closely related to members of the genus *Pseudomonas*, 3 strains to members of the genus *Aeromonas* and 1 strain closely related to members of genus *Bacillus*. The only three lipolytic strains that presented four hydrolytic activities (protease, amylase, DNAse and pullulanase) were isolated from the same sample. These three isolates (AE1B 20, AE1B 22 and AE1B 27) formed a clear branch together with species belonging to the genus *Aeromonas* (Table 5 and Fig. 3). Although AE1B 22 and AE1B 27 could belong to the same species, AE1B 20 clearly diverged from the other two, and it might be a different species.

Table S1. Hydrolytic activities of the 30 bacterial isolates capable of biofuel production. The closest relative of each isolate is indicated based on the phylogenetic reconstruction shown in Figure 3.

Sample	Closest relative	DNAse	Pullulanase	Xylanase	Amylase	Protease
**AE2B 29**	*Pseudomonas veronii*	-	-	-	-	+
**AE2B 30**		-	-	-	-	+
**AE2B 199**		+	-	-	+	-
**AE2B 222**		+	-	-	+	-
**AE2B 232**		+	-	-	+	-
**AE2B 263**		-	-	-	-	+
**AE2B 264**		-	-	-	-	+
**AE2B 332**		+	-	-	+	-
**AE2B 340**		+	-	-	+	-
**AE2B 130**	*Pseudomonas extremaustralis*	-	-	-	-	-
**AE2B 133**		-	-	-	-	-
**AE2B 134**		-	-	-	-	-
**AE2B 261**		-	-	-	-	-
**AE2B 259**	*Pseudomonas grimontii* CFML	-	-	-	-	-
**AE2B 85**	*Pseudomonas stutzeri*	-	+	-	+	-
**AE2B 120**		-	+	-	+	-
**AEDH 145**	*Acinetobacter*	-	-	-	-	-
**AE1B 89**	*Enterobacter ludwigii*	-	-	-	-	-
**AE1B 90**		-	-	-	-	-
**AE1B 92**		-	-	-	-	-
**AE1B 20**	*Aeromonas*	+	+	-	+	+
**AE1B 22**	*Aeromonas media*	+	+	-	+	+
**AE1B 27**		+	+	-	+	+
**AE1B 26**	*Bacillus simplex*	+	-	-	-	-

**AE1B 250**		+	-	-	-	-
**AE1B 35**	*Bacillus muralis*	-	+	-	-	+
**AE1B 28**	*Staphylococcus*	-	-	-	+	-
**AE1B 21**	*Terribacillus goriensis*[a] *T. saccharophilus*[a]	-	-	-	-	-
**AE2B 122**		-	-	-	-	-
**AE2B 131**		-	-	-	-	-

[a] Strains AE1B21, AE2B122 and AE2B131 showed the same % of similarity to both *T. goriensis* and *T. saccharophilus*
